# Factors Influencing Adherence to Adjuvant Endocrine Therapy After Breast Cancer Surgery

**DOI:** 10.1002/cnr2.2160

**Published:** 2024-08-19

**Authors:** Aina Johnsson, Anna von Wachenfeldt

**Affiliations:** ^1^ Department of Oncology and Pathology Karolinska Institute Stockholm Sweden; ^2^ Department of Oncology Södersjukhuset Stockholm Sweden; ^3^ Department of Clinical Science and Education, Södersjukhuset Karolinska Institute Stockholm Sweden

**Keywords:** adherence, anti‐hormonal side effects, breast cancer, life satisfaction

## Abstract

**Background:**

Women with newly diagnosed hormone receptor‐positive breast cancer are offered adjuvant endocrine therapy (AET). Despite the survival benefits of the therapy, a significant proportion of breast cancer patients do not adhere to the anti‐hormonal medication.

**Aims:**

The purpose of this study was to analyse demographic, social, psychological and treatment‐related factors influencing whether women diagnosed with early‐stage breast cancer were adherent to offered therapy.

**Materials and Methods:**

This was a long‐term retrospective, medical record study, supplemented with a questionnaire, including 81 women. Data from the Swedish Prescribed Drug Register were used to examine adherence. The women were followed for 5 years of offered AET.

**Results:**

Out of 81 women, 67 (83%) were adherent (hade taken out 80% or more of the recommended dose), 10 (12%) were Partially Adherent and 4 (5%) never accepted AET. At baseline, the Never‐Adherent group members were younger, more often considered themselves healthy and seemed much more satisfied with their lives. Baseline factors that positively affected adherence were satisfaction with the vocational situation (*p* = 0.023) and satisfaction with family life (*p* = 0.040). Cumulative musculoskeletal side effects were more frequently reported among women in the Adherent group than Partially Adherent women, after both 12 and 60 months (*p* = 0.018 and *p* = 0.011, respectively). There was also a significant difference in reported cumulative psychological side effects (*p* = 0.049) in disfavour of the Adherent group. Moreover, according to the questionnaire where the women retrospectively were asked which side effects, they experienced during the treatment period; sexual desire was significantly lower in the Adherent group (*p* = 0.0402) than in the Partially Adherent group.

**Conclusion:**

It is important to consider a woman's life situation, to support those who otherwise would not be able to complete AET and to help all women relieve side effects during AET. It should be investigated why some women did not start the recommended therapy.

## 1 Introduction

Women with hormone‐sensitive breast cancer (BC) are given adjuvant endocrine therapy (AET) to prevent relapses. Despite the survival benefits of AET [1], a significant proportion of BC patients—31% to 73%—do not adhere to the therapy [2, 3, 4, 5, 6].

AET is associated with several side effects that affect the quality of life, mainly menopausal symptoms and sexual dysfunction. Musculoskeletal side effects, depression, sleep disorders and gastrointestinal complaints have also been documented [5, 7, 8, 9, 10]. A Swedish study reported that BC patients, after surgery and before the start of AET, rated their life satisfaction lower in most domains compared with the general population. During AET, their life satisfaction deteriorated further [11].

In addition to side effects, socioeconomic status, age, language problems, co‐morbidity, pre‐diagnosis hormone replacement therapy, psychosocial and social factors, support from the health care providers, smoking and non‐participation in mammography screening have been shown to have an impact on patients' willingness to take prescribed medications [2, 3, 5, 12, 13, 14, 15, 16, 17, 18, 19]. In addition, non‐adherence to AET can occur because the benefits of the therapy are not obvious [20].

This study is a part of a larger project concerning experiences of anti‐hormonal side effects of AET, which started in January 2013 and involved three hospitals in the Stockholm region and one hospital in the Norrbotten region in Sweden, which included both focus group studies and retrospective data from 2002 onwards. The focus group studies addressed different aspects of how the women experienced their anti‐hormonal treatment [11, 21, 22, 23, 24, 25].

### 1.1 Purpose

The purpose of this study was to analyse demographic, social, psychological and treatment‐related factors influencing whether women diagnosed with early‐stage BC were adherent to AET offered after BC surgery.

### 1.2 Concepts

We consider a woman completely adherent if she took out 80% or more of the recommended dose of tamoxifen or aromatase inhibitor (AI) over 5 years, that is, for the entire recommended therapy duration. The 80% cut‐off was used, as it is the most commonly used definition of adherence to anti‐hormonal treatments [6, 26]. Adherence was calculated as the sum of defined daily doses divided by number of days recommended. Not completely adherent means not taking the prescribed amount of medicine, taking a break in AET on one or more occasions, discontinuing the therapy or refusing it in advance, or a combination of these. In this study, we divided the women into three categories based on the definition above: (1) those completely adhering to AET, labelled ‘Adherent’; (2) those who started AET, but did not achieve full adherence, labelled ‘Partially Adherent’ and (3) those who never started AET, labelled ‘Never‐Adherent’. The reason we distinguished between partial adherence and never‐adherence was that women who did not start AET did not suffer any AET side effects whatsoever.

### 1.3 Patients and Methods

All women at the oncology clinic at Södersjukhuset, Stockholm, who were recommended and given prescriptions for AET (tamoxifen or AI) after BC surgery and who fulfilled the inclusion criteria were eligible. Women diagnosed from December 2002 to April 2005, with a planned treatment period of 5 years were included retrospectively.

**FIGURE 1:**
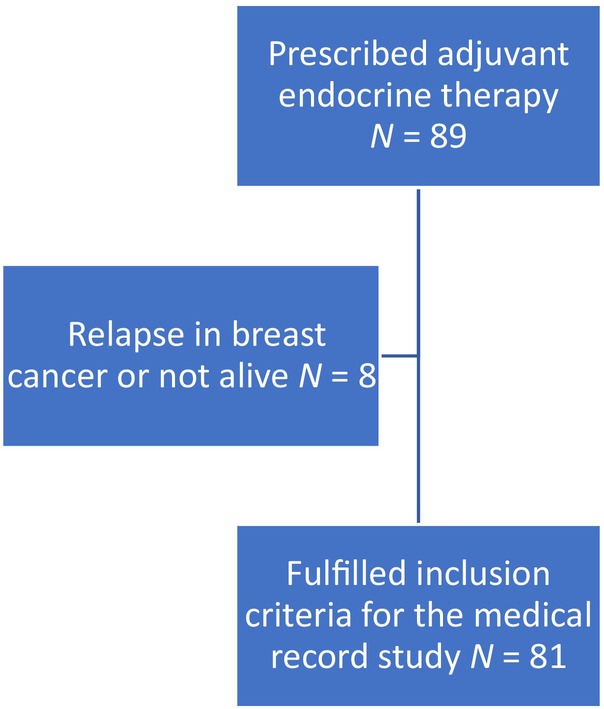
Flowchart—Medical record study.

Inclusion criteria were age 18–64 years and ability to read and understand Swedish. Deceased women and those with relapse were not included. The remaining 81 women met the inclusion criteria (see Figure 1).

### 1.4 Data Assessment Procedure

All medical records for the period 1–60 months after the date of AET initiation were retrospectively explored and all information about suspected side effects of AET was noted. All check‐ups took place at an oncology outpatient clinic. A template was developed for collection and classification of data. The template included the categories musculoskeletal, menopausal and psychological symptoms and sexual dysfunction disorders.

Treatment characteristics for chemotherapy, radiotherapy and breast surgery and data on prescribed AET were obtained from medical records. The women also completed several questionnaires.

**FIGURE 2:**
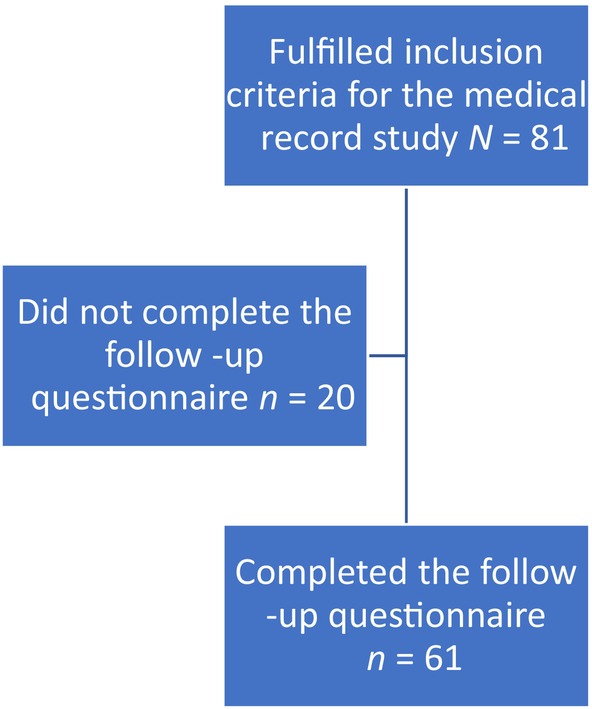
Flowchart—Follow up questionnaire.

A Follow‐up was performed 1½–2 years after the completion of 5‐year AET where we asked the women to reflect on the side effects they experienced during the treatment period (see Figure 2). We hypothesised that by this time the women would have recovered from the side effects of AET and would be able to reflect on the differences between a life with and without side effects. We based our assumption on that patients with BC generally recover over time [27, 28]. In an early study in the project on experiences of side effects of AET, we showed that patients had recovered in most LiSat‐11 domains 1½–2 years after therapy [11].

### 1.5 Factors Studied Included

1. Age, country of birth, marital status, having children or not, living with underage children, occupational code (Statistics Sweden) [25], self‐rated health and self‐rated health compared with others (questionnaires retrieved from Statistics Sweden) [29, 30].
2. Life satisfaction [31].

We used data from the Swedish Prescribed Drug Register to examine the women's adherence. The register encompasses all prescribed medicines dispensed in Swedish pharmacies since 1 July 2005. Information on each dispensed drug, the unique personal identification number of the person for whom the drug was prescribed and the date on which the prescription was dispensed were retrieved from the database. The register also includes information on active substances (using the Anatomical Therapeutic Chemical code), package size, number of packages and number of defined daily doses of each dispensed drug.

The majority (95%) of the women received most of their AET after the Swedish Prescribed Drug Register was introduced. If a woman retrieved prescriptions after the register was established on 1 July 2005, we assumed that she also took medicine before the register opened. The 67 women who, according to their medical records, started AET before the register was established retrieved prescriptions after the register started and were 100% compliant during the registered time. We have therefore chosen to list them as adherent.

### 1.6 Instruments

#### 1.6.1 LiSat‐11

The generic quality of life instrument *LiSat‐11* monitors self‐rated life satisfaction in 11 dimensions: life as a whole, vocational situation, financial situation, leisure situation, contact with friends, sexual life, activities of daily life, family life, partner relationship, somatic health and psychological health. For each item, the women rated their level of satisfaction on a 6‐point scale (1 = very dissatisfied, 2 = dissatisfied, 3 = rather dissatisfied, 4 = rather satisfied, 5 = satisfied, 6 = very satisfied). The instrument is well‐validated and normative data for Swedish women are available [31].

The LiSat‐11 questionnaire was sent to the women by post from baseline and every 3 months for 18 months.

#### 1.6.2 Anti‐Hormonal Side Effect Questionnaire

To compare the results from the medical records with the women's own experiences, a questionnaire addressing AET side effects were sent to the women by post between 1½ and 2 years after completion of AET. The questionnaire had the same design as the template in the medical records study. For each item, the women had to retrospectively state the level of the disorders during the therapy period on a 5‐point scale (1 = no problem, 2 = minor problem, 3 = moderate problem, 4 = severe problem, 5 = very severe problem).

### 1.7 Compliance With Ethical Standards

All procedures were in accordance with the 1964 Helsinki Declaration and its later amendments. The study was approved by the Research Ethics Committee South, with additional approval from the Regional Ethical Review Board in Stockholm.

Informed consent was obtained from all participants.

### 1.8 Statistical Methods

The dichotomous variables are described in tables, which show percentages within groups. Relationships between categorical variables were analysed using the *χ* ^2^ test where appropriate and Fisher's exact test where the *χ* ^2^ test was not appropriate. Logistic regression was used for analyses of more than one risk factor at a time. In the logistical regressions, the variable *Satisfied with family life* was dichotomised into <5 and (5 + 6) based on the response options in the LiSat‐11 scale. A rule of thumb is that when 20% or more of the expected frequencies in the cells of the 2 × 2 table are <5, the *χ* ^2^ test is not appropriate.

Continuous variables and categorical variables with ordered categories, as seen in LiSat‐11 and other rating scales, are presented with medians. Differences in these variables between the different Adherent groups were analysed using non‐parametric tests. The Mann–Whitney *U*‐test was used to test whether two predefined groups (Adherent versus Partially Adherent + Never‐Adherent; Adherent versus Partially Adherent; Adherent versus Never‐Adherent) had identical distributions. The Kruskal–Wallis one‐way analysis of variance by ranks was used in this analysis to compare the three predefined groups in order to test the null hypothesis that all predefined populations had identical distributions of the variable of interest.

## 2 Results

According to the Swedish Prescribed Drug Register, 67 (83%) out of 81 eligible women were Adherent, 10 (12%) were Partially Adherent and 4 (5%) were Never‐Adherent to AET, that is, never accepted the therapy. The predefined group Partially Adherent consisted of women who did not take out the prescribed amount of medicine, discontinued AET or both. The four women defined as Never‐Adherent appeared in their medical records to have expressed reluctance to start AET.

Demographic and social characteristics and self‐rated health at baseline, by group, are presented in Table 1. The majority, 55 women (68%), lived with a partner. In total, 66 (81%) women had children; 15 lived with at least one child under 18 years of age. Fifty‐one women rated their health as good or very good and 67 women rated their health as just as good as or better than that of others.

**TABLE 1:** Demographic and social characteristics, self‐rated health, and sense of coherence at baseline among 81 women treated with adjuvant endocrine therapy (AET).

	All the women *N* = 81	Adherent group *N* = 67	Partially Adherent group *N* = 10	Never‐Adherent group *N* = 4
	*n* (%)	*n* (%)	*n* (%)	*n* (%)
Age, years				
35–54	38 (47)	31 (46)	3 (30)	4 (100)
55–63	43 (43)	36 (54)	7 (70)	0 (0)
Living with husband/partner				
Yes	55 (68)	46 (69)	7 (70)	2 (50)
No	26 (32)	21 (31)	3 (30)	2 (50)
Children (not childless)				
Yes	66 (81)	57 (79)	6 (60)	3 (75)
No	15 (18)	10 (21)	4 (40)	1 (25)
Living with a child under 18 years of age				
Yes	15 (19)	14 (21)	1 (10)	0 (00)
No	66 (81)	53 (79)	9 (90)	4 (100)
Born in Sweden				
Yes	63 (78)	54 (81)	6 (60)	3 (75)
No	18 (22)	13 (19)	4 (40)	1 (25)
Self‐rated health				
Very good or good	51 (63)	40 (60)	7 (70)	4 (100)
Fair, poor, or very poor	30 (37)	27 (40)	3 (30)	0 (0)
Self‐rated health compared with others				
As good as or better	67 (83)	54 (81)	9 (90)	4 (100)
Poor or very poor	14 (17)	13 (19)	1 (10)	0 (0)

Treatment characteristics at baseline, by adherence groups, are presented in Table 2. Of the women who were adherent, two switched from tamoxifen to AI and four switched from AI to tamoxifen during the AET period (data not shown). Among the women who were Partially Adherent, no therapy changes occurred.

**TABLE 2:** Medical treatment characteristics at baseline among 81 women treated with adjuvant endocrine therapy (AET).

	All the women *N* = 81 *n* (%)	Adherent *N* = 67 *n* (%)	Partially Adherent *N* = 10 *n* (%)	Never‐adherent *N* = 4 *n* (%)
Chemotherapy				
Yes	26 (32)	23 (34)	2 (20)	1 (25)
No	55 (68)	44 (66)	8 (80)	3 (75)
Surgery				
Mastectomy	28 (35)	23 (34)	3 (30)	2 (50)
Breast‐conserving surgery	53 (65)	44 (66)	7 (70)	2 (50)
Radiotherapy (RT)				
RT to breast/chest wall and regional nods	15 (18)	14 (21)	1 (10)	0 (0)
RT only to breast parenchyma	48 (60)	39 (58)	7 (70)	2 (50)
No RT	18 (22)	14 (21)	2 (20)	2 (50)
Planned AET				
AI	41 (51)	36 (54)	3 (30)	2 (50)
Tamoxifen	40 (49)	31 (46)	7 (70)	2 (50)

Abbreviation: AI = aromatase inhibitor.

### 2.1 Results by Demographic and Social Factors at Baseline

None of the demographic characteristics, social characteristics or self‐rated health showed significance. It is noteworthy that a higher proportion of women born in Sweden was adherent to AET 86% compared with 72% among women born in other countries; this difference was not significant (*p* = 0.286; data not shown).

A lower proportion of women without children was adherent to AET, 67% compared with 86% among women with children. Again, the difference was not significant (*p* = 0.122; data not shown).

The Never‐Adherent group differed significantly from both the Adherent and the Partially Adherent groups. All four women in the Never‐Adherent group were in the youngest age category, three of four were born in Sweden and three had children, but none lived with underage children. All four rated their own health as good and perceived it to be as good as or better than that of others (Table 1).

### 2.2 Results by Life Satisfaction at Baseline

It can be noted that we used LiSat‐11 at baseline and then every 3 months for 18 months. The small changes in life satisfaction noted during the follow‐up period were similar in the three predefined groups. We have therefore chosen to report the baseline data as the response frequency was highest then.

**FIGURE 3:**
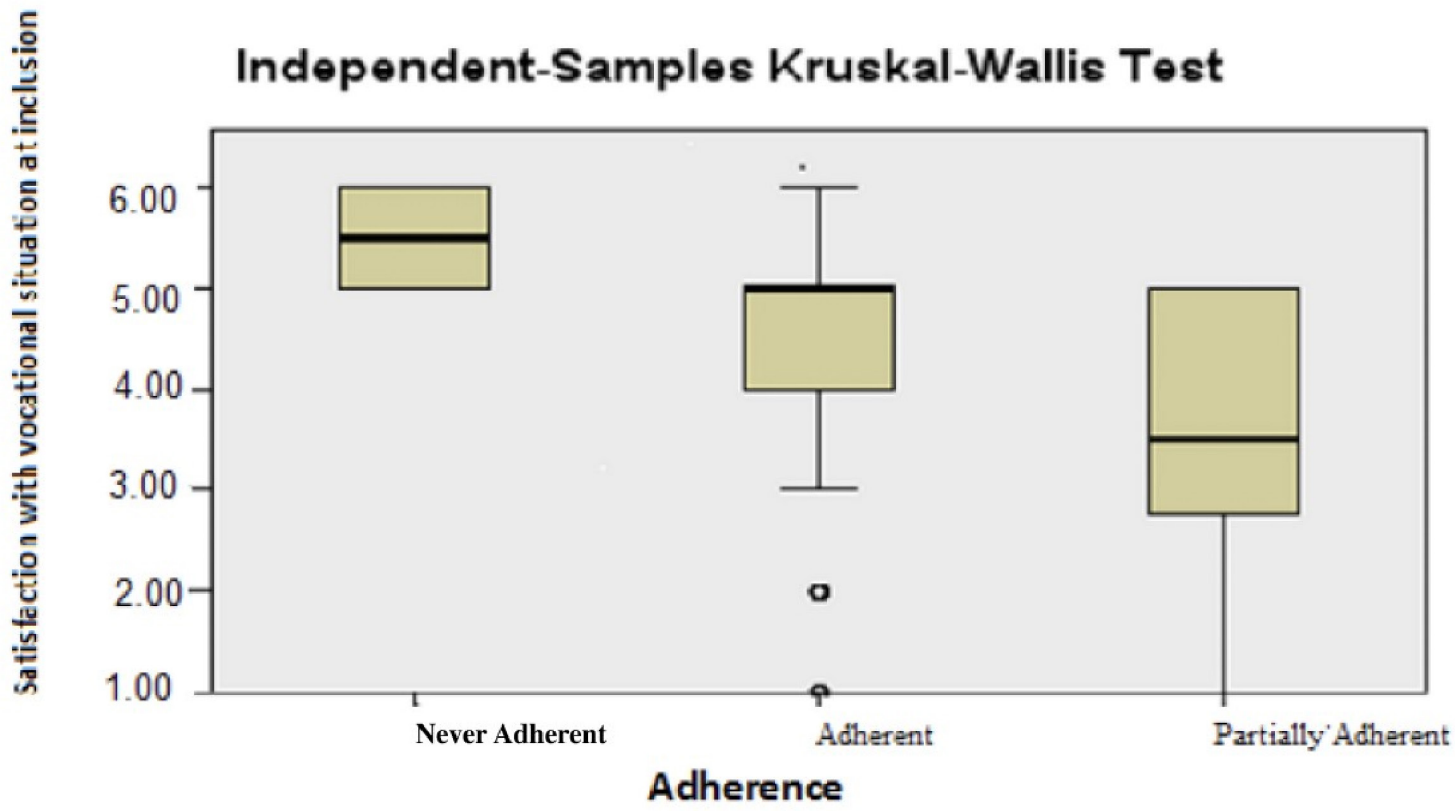
Satisfaction with vocational situation at baseline.

**FIGURE 4:**
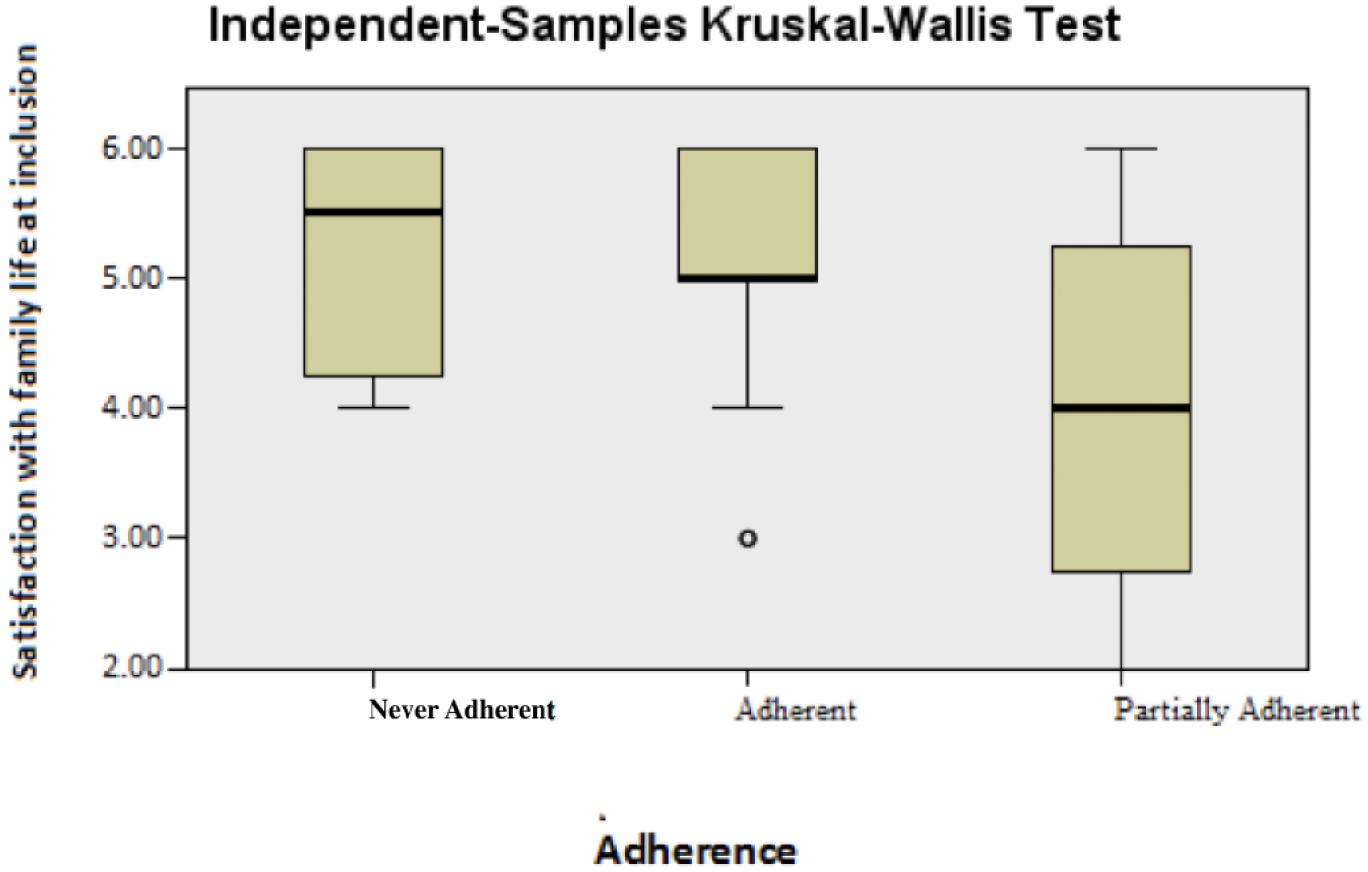
Satisfaction with family life at baseline.

Regarding the two variables *Satisfaction with the vocational situation* and *Satisfaction with family life* at baseline, the differences between the groups were significant. For both these variables, the Never‐Adherent group had the highest satisfaction, while women in the Partially Adherent group were the least satisfied. The largest statistical spread was shown for vocational situation (Figures 3 and 4 respectively Table 3). When the two groups Never‐Adherent and Partially Adherent were merged into one and compared with the Adherent group, the difference was no longer significant.

**TABLE 3:** Life satisfaction at baseline in the predefined groups.

	*N*	Median	*p*
Vocational situation			0.023
Adherent group	67	5.00	
Partially Adherent group	10	3.50	
Never‐Adherent group	4	5.50	
**Total**	**81**		
Family life			0.040
Adherent group	67	5.00	
Partially Adherent group	10	4.00	
Never‐Adherent group	4	5.50	
**Total**	**81**		

### 2.3 Results by Treatment Characteristics at Baseline

No significant differences were detected between those who were adherent and those who were not regarding chemotherapy (*p* = 0.53), radiotherapy to breast/chest wall and regional nodes (*p* = 0.45) or type of surgery (*p* = 1.00). Among those who received Al, a higher proportion was adherent to AET, 90% compared with 80% among those receiving tamoxifen; this difference was not significant (*p* = 0.225; data not shown).

### 2.4 Adherence by Cumulative Side Effects During Treatment Reported in the Medical Records

A total of 1000 notes in the medical records were analysed, covering the 5 years of planned AET for all 81 included women, with a mean of 6.2 notes (range 4–12) per woman. Below and in Tables 4 and 5, data are reported from the Adherent and Partially Adherent groups only, because the Never‐Adherent group was never exposed to AET.

**TABLE 4:** Cumulative number of side effects reported in the medical records after 12 months among 77 women who were adherent or Partially Adherent to adjuvant endocrine therapy (AET).

Type of side effects	*n*	Median	Minimum	Maximum	*p*
After 12 months					
Menopausal/sexual dysfunction					0.969
Adherent group	67	1	0	12	
Partially Adherent group	10	1.5	0	10	
**Total**	**77**				
Musculoskeletal					0.018
Adherent group	67	0	0	7	
Partially Adherent group	10	0	0	1	
**Total**	**77**				
Psychological					0.066
Adherent group	67	0	0	9	
Partially Adherent group	10	0	0	0	
**Total**	**77**				

**TABLE 5:** Cumulative number of side effects reported in the medical records after 30 months among 77 women who were adherent or partially adherent to adjuvant endocrine therapy (AET).

Type of side effects	*n*	Median	Minimum	Maximum	*p*
After 60 months					
Menopausal/sexual dysfunction					
Adherent group	67	2	0	14	
Partially Adherent group	10	2	0	10	1.000
**Total**	**77**				
Musculoskeletal					
Adherent group	67	1	0	11	
Partially Adherent group	10	0	0	1	0.011
**Total**	**77**				
Psychological					
Adherent group	67	0	0	11	
Partially Adherent group	10	0	0	0	0.049
**Total**	**77**				

After both 12 and 60 months, musculoskeletal side effects were significantly more frequently reported among women in the Adherent group than in Partially Adherent group. At 60 months, there was also a significant difference in reported psychological side effects in disfavour of the Adherent group. Regarding menopausal side effects and sexual dysfunction, no significant difference was seen at either 12 or 60 months.

### 2.5 Retrospectively Self‐Reported Anti‐Hormonal Side Effects

A total of 60 women completed the side effect questionnaire. Sexual desire was significant lower in the Adherent group (*p* = 0.0402) than in the Partially Adherent group. For the other studied menopausal side effects, the *p*‐value range was 0.51–0.95.

## 3 Discussion

The purpose of this study was to describe the differences and similarities between treatment‐related, demographic, social and psychological factors in women who were adherent, Partially Adherent, or never accepted AET after BC surgery. Adherence to AET was not affected by the type of treatment, such as chemotherapy or radiotherapy to breast/chest wall and regional nodes, which would indicate a higher severity of BC [32]. We found that patient‐related factors affected adherence. The women in the Partially Adherent group were less satisfied with family life and vocational situation than women in the Adherent group. Women who had children were numerically more likely to complete AET, but this difference was not significant (*p* = 0.122). Neither the medical record review nor the retrospective follow‐up questionnaire showed any significant differences regarding menopausal side effects between the groups. However, the Adherent group reported more side effects relating to musculoskeletal and psychological disorders. In the follow‐up, more women from the Adherent group than from the Partially Adherent group experienced sexual dysfunction. These results are likely due to the fact that the women in the Adherent group were exposed to a longer period of AET than those in the Partially Adherent group. The results may also indicate that the women in the Adherent group had higher motivation to complete AET and therefore requested help to improve their ability to do so.

At baseline women in the Partially Adherent group were less satisfied with family life, vocational situation and partner relationship than women in the Adherent group. Personal social support [15] and quality of life [14] are known to be factors that affect adherence to AET. In a Swedish study conducted in 20–63‐year‐old women shortly after BC surgery it was shown that having children, being in work, having emotional and informational social support and having good physical and emotional functioning were positively associated with satisfaction with life as a whole [33]. These results may indicate that those who do not complete therapy have fewer, or less supportive, relationships. This may cause considerable vulnerability, which may in turn result in having more difficulties adapting to the side effects of AET.

In the medical record study, we have shown that the higher the number of cumulatively reported psychological problems after 60 months, the more adherent were the women. This may indicate that anxiety over experiencing a BC relapse results in strong motivation to complete AET. It may also indicate that the women who were not fully Adherent valued their life situation less and therefore were less worried about a BC relapse compared with the adherent women. Another explanation could be that lower life satisfaction is a reflection of the underlying challenges—either health related or social/financial barriers, which may also make adherence to medication more challenging. The question is what price, in the form of side effects, a woman who does not value her life situation especially highly is ready to pay to at best improve her chances of survival.

The Never‐Adherent group differed significantly from both the Adherent group and the Partially Adherent group. It is difficult to draw any firm conclusions based on this finding as the Never‐Adherent group consisted of only four women. However, it can be noted that women in the Never‐Adherent group were younger than those in the Partially Adherent group and considered themselves as healthy as others. In addition, the four women were in an age range where the normal menopause generally is many years ahead and where AET therefore risked leading to a significantly reduced quality of life.

It is also noteworthy that adherence to AET was not affected by type of treatment, such as chemotherapy or radiotherapy to breast/chest wall and regional nodes, despite this indicating a greater BC severity [32].

### 3.1 Strengths and Limitations

Strengths of this study that both the external and internal drop‐out rates were low. The authenticity of the medical characteristics was guaranteed by their being collected from medical records. Further, we used well‐known instruments (surveys from Statistics Sweden [25, 26], as well as LiSat‐11) which have previously been used to measure different aspects of being diagnosed with cancer. These instruments have never previously been used to measure adherence to AET. Therefore, we were able to find previously unknown factors that predicted adherence to AET.

One limitation of this study is the small sample. A larger study sample might have revealed other possible associations between the studied factors and adherence to AET. Another limitation is that only Swedish‐speaking women were included, meaning that our results may not be representative of women who do not speak Swedish. One could argue that the data were somewhat outdated. However, AET has not been changed significantly in recent decades [8, 34].

### 3.2 Conclusion

This study has shown that there is a relationship between how women with BC experience their life and their adherence to prescribed AET. It is therefore important to note a woman's life situation when prescribing AET, in order to support women who may otherwise not be able to complete the therapy. Interventions aimed at enhancing life satisfaction should be tested as part of improving adherence.

It deserves to be pointed out that research is currently being conducted that addresses the possibility of individualising AET at the molecular level and thereby reducing the negative consequences of the therapy. Such adaptation will likely contribute to more patients being able to complete their treatment [35, 36].

In the ongoing research on adherence to AET, it should also be noted if women who do not accept AET at all differ significantly from those who interrupt or discontinue AET. It would be important to study the reasons why these women do not accept the recommended AET.

## Author Contributions

**Aina Johnsson:** investigation, writing – original draft, methodology, validation, visualization, project administration, resources, funding acquisition, conceptualization. **Anna von Wachenfeldt:** conceptualization, investigation, writing – original draft, methodology, validation, project administration, resources, funding acquisition, visualization.

## Conflicts of Interest

The authors declare no conflicts of interest.

## Data Availability

The data are available on request from the corresponding author. The data are not publicly available due to privacy or ethical restrictions.
